# Isolated extravascular implantable cardioverter-defibrillator infection without leadless pacemaker involvement: A unique dual-device case report

**DOI:** 10.1016/j.hrcr.2026.01.028

**Published:** 2026-02-11

**Authors:** Elodie Deschamps, Marta Garcia-Montero, Alexandre Raymond-Paquin, Blandine Mondésert

**Affiliations:** Department of Medicine, Montreal Heart Institute, Université de Montréal, Montreal, Quebec, Canada

**Keywords:** EV-ICD, Leadless pacemaker, Device infection, Micra, Cardiac sarcoidosis, Case report

**Key Teaching Points**

▪ Extravascular implantable cardioverter-defibrillator infections can remain anatomically compartmentalized, allowing selective extraction, while preserving a co-implanted leadless pacemaker when systemic involvement is excluded.
▪ Fluorodeoxyglucose– tomography imaging is a valuable tool for accurately localizing device infections and guiding management strategies in dual-device systems.
▪ Leadless pacemakers have a low susceptibility to infection, supporting their preservation in cases of nonsystemic codevice infections.

## Introduction

Implantable cardioverter-defibrillators (ICDs) have been central in the prevention of sudden cardiac death for more than 3 decades.^1^, ^2^, ^3^ Despite their proven efficacy, traditional transvenous ICD (TV-ICD) systems carry substantial procedural and long-term risks, primarily owing to the presence of intravascular leads.^4^^,^^5^ These complications mainly include lead malfunction, venous thrombosis, valvular injury, and infection. Although relatively infrequent (0.6%–1.3%), TV-ICD infections often require complete system removal and are associated with significant morbidity and mortality.^6^^,^^7^ To address these limitations, alternative extravascular systems have been developed. The subcutaneous ICD (S-ICD) avoids transvenous access and reduces many lead-associated complications.^8^ However, its lack of pacing capabilities limits its use in patients with bradyarrhythmias or those who may benefit from antitachycardia pacing (ATP). In addition, the greater distance from the heart necessitates higher shock energy with a subsequent larger device size and reduced battery longevity. More recently, extravascular ICD (EV-ICD) systems using substernal lead positioning have emerged, offering defibrillation and ATP with energy thresholds and battery longevity comparable with TV-ICDs,^9^, ^10^, ^11^ while maintaining a completely extravascular configuration. For patients who require chronic pacing, the combination of leadless pacemakers with either S-ICD or EV-ICD systems is increasingly reported, offering the benefit of avoiding transvenous leads altogether.^12^^,^^13^ Leadless pacemakers are associated with an extremely low, almost anecdotal, infection rate, with only rare cases reported in the literature despite widespread use.^14^, ^15^, ^16^ Although each system individually has a lower risk of infection, data on dual-device implantations remain scarce, particularly regarding cross-contamination, systemic dissemination, and the need for full vs partial system removal. In this context, we report a unique case of localized EV-ICD infection without involvement of a coimplanted Micra leadless pacemaker, and we discuss its clinical and management implications.

## Case report

**Figure 1:**
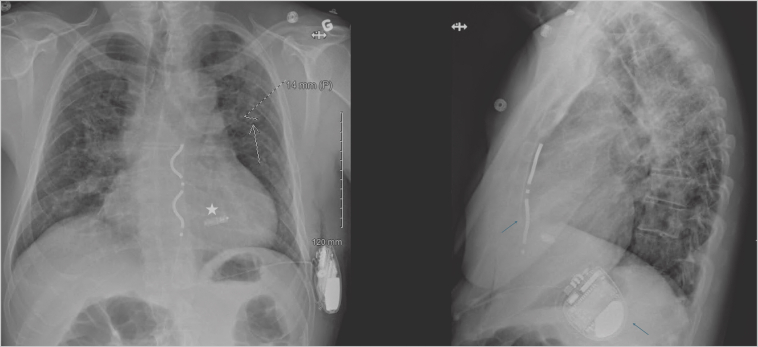
Face and lateral chest radiograph showing the Micra pacemaker (*whitestar*) and the substernal extravascular implantable cardioverter-defibrillator system (*blue arrows*).

**Figure 2:**
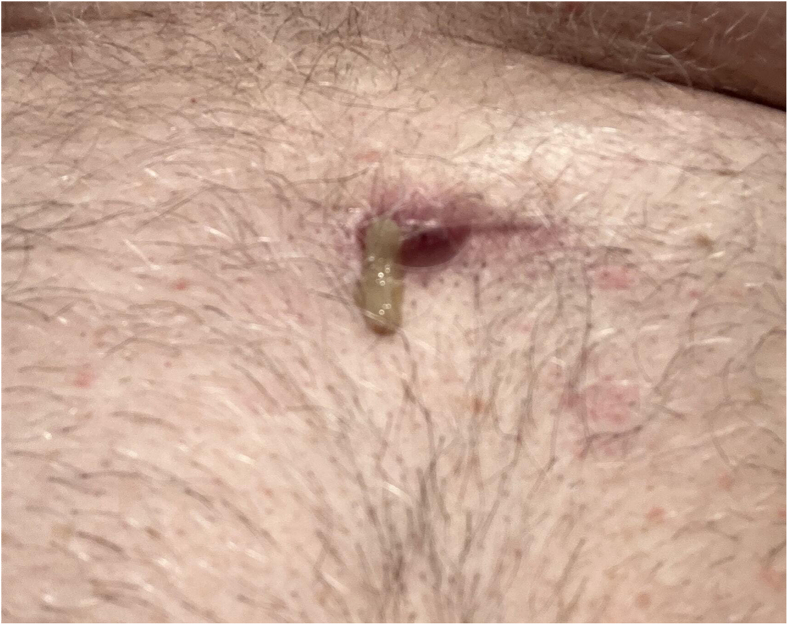
Local erythema and purulent drainage over the intermuscular generator pocket incision.

**Figure 3:**
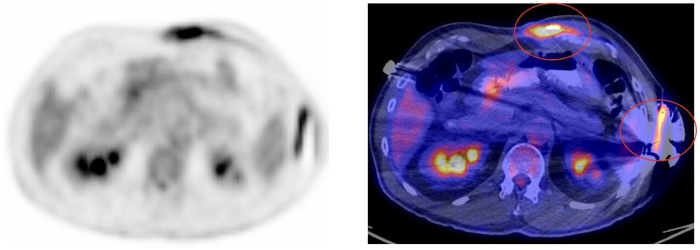
Attenuation-corrected fluorodeoxyglucose–positron emission tomography/computed tomography image showing focal radiotracer uptake localized to the extravascular implantable cardioverter-defibrillator generator and the distal portion of the lead (*red circles*).

A 62-year-old man with a medical history of pulmonary sarcoidosis treated with immunotherapy and corticotherapy presented several years earlier with syncope. At that time, his electrocardiogram revealed a second-degree atrioventricular block and a left bundle branch block. He also had a known history of bicuspid aortic valve, atrial fibrillation, and dyslipidemia. Further evaluation, including fluorodeoxyglucose–positron emission tomography (FDG-PET) imaging, demonstrated increased uptake in the interventricular septum, suggestive of possible cardiac sarcoidosis. A transvenous dual-chamber ICD was subsequently implanted via a left subclavian approach. Recently, in May 2024, the patient was admitted after experiencing multiple inappropriate shocks owing to noise. Device interrogation also revealed intermittent loss of capture, consistent with a lead fracture. Lead extraction was performed, and a new ICD lead was implanted in a septal position. In December 2024, he presented with dyspnea. Transthoracic echocardiography showed a large pericardial effusion with early signs of cardiac tamponade. Cardiac computed tomography demonstrated migration of the ICD lead into the pericardial space, confirming lead perforation. The right ventricle lead was removed, and a new one was implanted in an apical position. Within 2 days, the patient developed a recurrent pericardial effusion requiring pericardiocentesis. Considering these repeated complications related to transvenous leads, a leadless approach was chosen. The perforating right ventricle lead was removed, and a Micra AV2 (Medtronic) leadless pacemaker was implanted. An EV-ICD system (Medtronic) was implanted in a second procedure, with the lead placed substernally and the generator positioned in an intermuscular pocket (Figure 1). In February 2025, the patient noted localized inflammation over the intermuscular pocket accompanied by purulent exudate (Figure 2). Blood cultures remained negative without any clinical evidence of sepsis. A wound culture was positive for *Pseudomonas aeruginosa*. FDG-PET imaging confirmed active infection involving the EV-ICD generator and the distal portion of the substernal lead (Figure 3). Importantly, the Micra device showed no signs of infection or inflammatory uptake on imaging. The patient was started on oral antibiotic therapy, and the EV-ICD system was removed. Intraoperative cultures confirmed the presence of *Pseudomonas aeruginosa* and *Staphylococcus hominis.* Given the absence of active sarcoidosis under immunosuppressive therapy, the lack of appropriate ICD therapies since the first ICD implant, the absence of initial histologic confirmation of cardiac involvement, and the series of device-related complications, it was decided not to reimplant a defibrillator at this time (life vests are not supported in Canada). At the most recent follow-up, the patient remained asymptomatic, with complete wound healing. Interrogation of the Micra device showed stable function, with a low ventricular pacing burden (<20%) and stable electrical parameters. A detailed chronological overview of his subsequent procedures and complications is presented in Table 1.

**Table 1:** Chronological timeline of events

Date	Event
July 2021	Syncope in a 62-y-old man with suspected cardiac sarcoidosis → dual-chamber transvenous ICD implanted (left subclavian approach)
May 2024	Inappropriate shocks owing to lead noise + intermittent loss of capture → lead extraction and septal reimplantation
December 2024	Pericardial effusion → lead removed + new lead placed in an apical position
December 2024 (2 d later)	Recurrent tamponade after procedure → Micra AV2; EV-ICD implanted 7 d later
February 2025	Purulent drainage over EV-ICD site + FDG-PET confirms infection of EV-ICD components only (no Micra involvement) → EV-ICD explanted, Micra preserved
May 2025	Asymptomatic, wound healed, Micra functioning normally, no ICD reimplantation to date; reimplantation to be reconsidered after complete healing

EV-ICD = extravascular implantable cardioverter-defibrillator; FDG-PET = fluorodeoxyglucose–positron emission tomography; ICD = implantable cardioverter-defibrillator.

## Discussion

To the best of our knowledge, this is the first published report of an isolated EV-ICD infection with complete sparing of a coimplanted Micra leadless pacemaker. This case illustrates the anatomic and functional separation of the 2 systems, with implications for infection management and device retention. EV-ICDs offer a promising alternative to transvenous systems, delivering effective defibrillation and ATP through a substernal lead, while remaining fully extravascular. In the pivotal EV-ICD trial, the device met safety and efficacy end points, with ATP success in 70% of episodes and a 92.6% freedom from major complications.^9^ At 3 years, long-term data remained favorable with 77% ATP success and 89% complication-free survival.^10^ Infection rates in EV-ICD trials were 2.5% at 3 years, and only 2.2% required device explantation.^10^ None progressed to mediastinitis or systemic infection. These results are comparable with those observed with the S-ICD in large registries, where the incidence of S-ICD–related infections varies between 0.9% and 3.3%.^13^^,^^17^^,^^18^

This patient experienced several complications related to transvenous leads, including recurrent pericardial effusions and lead dysfunction, explaining the switch to a fully extravascular and leadless strategy. Interestingly, the third lead, which ultimately perforated, had been implanted in a septal position, a location generally considered at a lower risk of perforation. This unexpected event highlights the patient’s apparent susceptibility to lead-related complications and supports the rationale for avoiding further transvenous approaches.

In our case, FDG-PET imaging played a central role in our decision making by clearly localizing the infection to the EV-ICD components without evidence of extension. This observation aligns with preclinical findings, which have demonstrated a tendency for infections involving extravascular systems to remain anatomically compartmentalized. In a large animal study, 4 of 13 animals developed EV-ICD–related infections, none of which led to systemic involvement. 1 animal with a coimplanted transvenous CRT-D developed septicemia and endocarditis, whereas the EV-ICD components remained unaffected.^19^

The absence of FDG uptake around the Micra device was particularly notable. Its small profile, intramyocardial fixation, and limited exposed surface area likely contribute to its lower susceptibility to infection.^20^ These features may explain why the device remained uninvolved despite an active infection of the EV-ICD system.

Although some reports suggest that localized infections may be managed conservatively without explantation, we opted for complete removal of the system given the recent implantation and the infection spread to the substernal space. The extraction was performed without complication via simple traction. Current international guidelines do not yet provide specific recommendations for managing infections involving dual extravascular and leadless cardiac devices.^7^ Future consensus documents should address selective extraction algorithms in such anatomically separated systems. Moreover, questions remain regarding the feasibility and safety of removal in chronic implants. In fact, a multicenter analysis of EV-ICD extraction found high success rates for devices implanted under 36 months, but 1 failure occurred with a 58-month-old system owing to xiphoid adhesions and lead fracture associated with the use of a mechanical sheath in a noncoaxial position.^21^

This case further raises the issue of individualized risk assessment for reimplantation. In our patient, despite the absence of histologic confirmation of cardiac sarcoidosis, the combination of atrioventricular block, abnormal FDG-PET uptake, and concomitant pulmonary sarcoidosis fulfills several major criteria supporting a probable diagnosis of cardiac sarcoidosis according to recent diagnostic algorithms.^22^ Nevertheless, given the absence of sustained ventricular arrhythmias and a history of multiple device-related complications, conservative management with no ICD replacement was initially favored. Reimplantation of a nontransvenous device (either an S-ICD or an EV-ICD) will be reconsidered after complete healing of the pocket. An S-ICD will represent a reasonable option in this patient if screening confirms eligibility, whereas an EV-ICD would be reconsidered if ATP becomes necessary in the future. Importantly, the largest published multicenter analysis has shown that repeat substernal tunneling is technically feasible in most cases, even after device-related infections, with high procedural success rates. In our patient, the relatively recent implantation makes the likelihood of severe adhesions or fibrosis low.^21^

## Conclusion

This case highlights that EV-ICD infections can develop independently without affecting coimplanted leadless pacemakers. Advanced imaging with FDG-PET can help delineate infection extent and guide selective device management. Our experience supports the feasibility of compartmentalized explantation and suggests that, in appropriate cases, the leadless system may be safely preserved. Further studies are needed to inform evidence-based strategies for infection control in patients with EV-ICD and dual-device systems.

## Disclosures

Dr Marta Garcia-Montero was supported by a research grant from the Alfonso Martin Escudero Foundation. Dr Alexandre Raymond-Paquin receives speaker fees from Medtronic. Dr Blandine Mondésert is a presenter, consultant, and advisory board member for Medtronic. Dr Elodie Deschamps has no conflicts of interest to disclose.
